# Determinants of Dengue Serotype Shifts: A Narrative Multifactorial Perspective

**DOI:** 10.3390/v18060683

**Published:** 2026-06-18

**Authors:** Jeyanthi Suppiah, Sakshaleni Rajendiran, Siti Aishah Rashid, Nurulhusna Ab Hamid, Murni Maya Sari Zulkifli, Rozainanee Mohd Zain

**Affiliations:** 1 Virology Unit, Infectious Disease Research Centre, Institute for Medical Research, National Institutes of Health, Ministry of Health, Shah Alam 40170, Malaysiarozainanee@moh.gov.my (R.M.Z.); 2 Environmental Health Research Centre, Institute for Medical Research, National Institutes of Health, Ministry of Health, Shah Alam 40170, Malaysia; sakshaleni@moh.gov.my (S.R.);; 3 Entomology Unit, Infectious Disease Research Centre, Institute for Medical Research, National Institutes of Health, Ministry of Health, Shah Alam 40170, Malaysia

**Keywords:** dengue virus, serotype, herd immunity, outbreak, serotype shift

## Abstract

Dengue Virus (DENV) circulates as four antigenically distinct serotypes whose dominance fluctuates over time in many endemic regions, a phenomenon known as serotype shift that is frequently associated with large outbreaks and increased disease severity. This review, through a synthesis of epidemiological, virological, immunological, entomological, and environmental evidence, observes that serotype shift likely arises from the interaction of multiple determinants rather than solely from viral evolution, with population immunity playing a central role. The accumulation of serotype-specific herd immunity, together with short-lived cross-protection and Antibody-Dependent Enhancement (ADE), reshapes population susceptibility and creates ecological space for heterologous serotypes with higher transmission potential. The synthesis of global dengue studies indicates that these immune dynamics interact with viral genetic diversity, vector competence, climate variability, and human factors such as demography, socioeconomic status, population density and mobility to drive cyclical and sometimes abrupt changes in serotype dominance. Notably, the review indicates that serotype changes often precede or coincide with more clinical severity and patterns of outbreaks, with direct implications for the process of forecasting outbreaks, vaccine performance, and preparedness to respond with appropriate health measures. On the whole, this review confirms the opinion that the change of dengue serotype occurrence becomes a consequence of interconnected biological and ecological processes involved in the transmission of dengue serotype shifts in hyperendemic areas.

## 1. Introduction

Dengue fever is a mosquito-borne viral disease with an estimated 390 million infections occurring annually, of which approximately 96 million manifest clinically [1]. The disease is caused by dengue virus (DENV), a member of the *Flaviviridae* family, existing as four serotypes, capable of producing results of asymptomatic infections to severe disease, which can be life-threatening.

One characteristic of the epidemiology of dengue in endemic countries is periodic replacement of the most common circulating serotype, otherwise known as serotype shift or serotype replacement. These changes can have great consequences for the health of the population, frequently accompanied by an increase in the number of cases and progression in disease severity. The herd immunity at the population level has conventionally been associated with serotype shifts whereby the previously dominant serotype would inhibit its spread, and other serotypes characterized by lower immunity to the infection would proliferate [2]. Nevertheless, these processes cannot be considered only as immunologically based dynamics; they result from a complex interplay of various factors. Past trends of serotype dominance with cyclical patterns have been observed in endemic regions, often associated with more frequent or more severe outbreaks. Consequently, these have led to the realization of the need to study the dynamics of serotype shifts in order to predict the occurrence of epidemiological transitions. Accordingly, this review presents a narrative synthesis of existing knowledge, epidemiological data on the occurrence of serotype shifts across regions, the multifactorial drivers of these replacements, and their implications for control and vaccine strategies.

**Figure 1:**
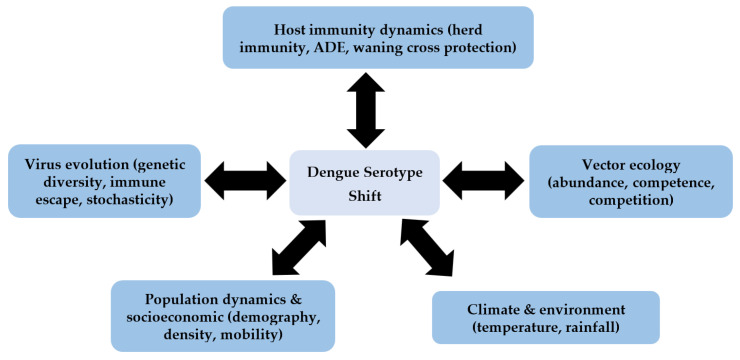
Conceptual framework illustrating the multifactorial drivers of dengue serotype shifts and their interactions across temporal scales. Arrows indicate hypothesized causal pathways and interactions.

We suggest a framework of systems-level interactions in which changes in DENV serotypes result from interplay of five key mechanisms: host immune dynamics, virus evolution, vector ecology, climate and environment and population dynamics and socioeconomic factors (Figure 1). The relative process of these drivers acts at various time scales. Immunity dynamics, viral factors and vector population typically evolve over multi-year periods, shaped by cumulative exposure and demographic turnover. In contrast, climate variability act over seasonal timescales, modulating transmission intensity. Moreover, population dynamics and socioeconomic factors function at intermediate timescales, facilitating the introduction of new serotypes or genotypes. Interactions among these drivers are likely complex and non-proportional, such that their combined effects may only promote serotype replacement once critical epidemiological or ecological thresholds are reached, allowing a newly introduced or previously suppressed serotype to become dominant.

## 2. The DENV and Its Antigenic and Genetic Diversity

DENV exists as four genetically and antigenically distinct serotypes: DENV-1, DENV-2, DENV-3, and DENV-4. The major determinants of antigenicity are located on the envelope (E) glycoprotein, particularly domain III, which induces type-specific neutralizing antibodies. On the other hand, more conserved epitopes in domain II and the pre-membrane (prM) protein often stimulate cross-reactive yet weakly neutralizing antibodies. Additionally, the non-structural protein 1 (NS1) is an important secreted antigen used in diagnostics and implicated in immune cross-reactivity with host proteins. Notably, antigenic diversity also exists within serotypes at the genotype level, wherein neutralization titers reveal subtle antigenic variation driven by a small number of key mutations. This demonstrates that antigenic evolution of dengue is ongoing, albeit less pronounced than inter-serotype differences [3].

Although they belong to the same viral species, these serotypes differ genetically by approximately 30.0–35.0% in the amino acid sequence of the E protein, the primary target of virus-neutralizing antibodies [4]. Within each serotype, further genetic diversity has led to the classification of multiple genotypes, typically defined by 6.0–8.0% nucleotide variation in the E gene. These genotypes often exhibit distinct geographical distributions [5].

DENV-1 was first identified in French Polynesia and Japan in 1943, followed by outbreaks in Hawaii in 1944 [6]. DENV-1 comprises five recognized genotypes (I–V) (Figure S1), with a sixth genotype proposed in 2016 based on a divergent isolate from Brunei [7], although this has not yet been confirmed by subsequent studies. Meanwhile, DENV-2 was first reported in 1944 in Papua New Guinea and Indonesia. Since the early 1960s, countries such as Malaysia and Thailand have documented continuous DENV-2 transmission, followed by widespread reporting from neighboring nations, including Indonesia, China, and India [6]. DENV-2 is regarded as the most genetically diverse serotype and is classified into six genotypes (Figure S2), formerly known as Asian I, Asian II, American, American/Asian, Cosmopolitan, and Sylvatic [8]. Furthermore, the origin of DENV-3 can be traced to 1953, when it was first reported in the Philippines and Thailand [6]. Its presence gradually expanded across Asia, with Thailand and Malaysia often serving as a key hotspot (Figure S3), DENV-3 consists of five genotypes (I–V) [9]. DENV-4, much like DENV3, was initially reported in Thailand and the Philippines [6]. Since 1978, it has been detected almost annually in Sri Lanka. In the Americas, DENV-4 did not emerge until after 1981. So far, four genotypes (I–IV) have been described (Figure S4), although their distribution worldwide is underreported, and recent findings are limited.

The antigenic and genetic differences among dengue serotypes have significant implications for the transmission processes and potential risks of outbreaks especially in hyperendemic settings. Periodic shifts in dominant serotypes occur approximately every three to five years in many dengue-endemic countries [10]. These shifts often precede large-scale epidemics and are associated with changes in disease severity, particularly when the newly dominant serotype encounters a population with limited immunity. In line with this, introducing a novel genotype with increased epidemic potential into a region can lead to explosive outbreaks.

## 3. Defining Dengue Serotype Shift: Concepts and Terminology

The term serotype shift refers to the phenomenon in which the predominant circulating DENV serotype changes over time within a given population or geographic region. This shift can occur gradually or abruptly and is often associated with changes in disease patterns, particularly in terms of incidence rates and the severity of clinical manifestations. However, understanding serotype shift requires clarification of key terms and concepts that underpin this process.

It is important to distinguish between DENV serotype shift and genotype shift, as both contribute to the epidemiology of dengue but operate through different mechanisms. A serotype shift involves replacing one of the four antigenically distinct serotypes (DENV-1, DENV-2, DENV-3, or DENV-4) with another. A genotype shift, on the other hand, is a change in a genetic variant (genotype) of a certain serotype by another. Although both serotype and genotype changes can contribute to dengue spread and severity, serotype changes can directly relate to immunity dynamics. In contrast, genotype changes can indicate viral evolution and adaptability to new environments or vectors [11,12].

## 4. Epidemiological Evidence of DENV Serotype Shifts

The epidemiological importance of serotype shifts is significant, as they may influence the occurrence of dengue outbreaks and their clinical consequences. For example, in Asia, the re-emergence of DENV-2 in some regions after a long period of DENV-1 dominance has been associated with more severe outbreaks [13,14]. Similarly, in Singapore, analysis of monthly dengue case trends from 2005 to 2008 revealed a shift in the predominant serotype from DENV-1 to DENV-2 in early 2007. This transition coincided with a clade replacement within DENV-2, which precipitated a major outbreak [15]. DENV-2 subsequently remained the dominant circulating serotype until 2013, when DENV-1 re-emerged and rapidly displaced it, once again triggering a large-scale outbreak [16]. In Bangladesh, following years of DENV-1 or DENV-2 circulation, DENV-3 became dominant in 2019, coinciding with the country’s then-largest outbreak. In that sense, DENV-3 has continued to play a major role in subsequent surges [17]. The Lao People’s Democratic Republic reported a striking upsurge of DENV-4 from 2016 to 2018, with the spread at the country level following two large epidemics, predominated by DENV-3 and DENV-1, respectively, in 2012–2013 and 2015 [18]. In Thailand, DENV-1 predominated in 2004, but by 2007 DENV-4 became the main serotype, and in 2008 DENV-1 was rapidly displaced [19]. This indicates that the epidemiology of dengue is dynamic, with the prevalence of a serotype often changing rapidly and often determining the pattern of outbreaks.

In Africa, recent studies indicate that all four dengue virus serotypes circulate during both epidemic and interepidemic periods, with DENV-2 being the predominant serotype before the 2000s. However, this pattern has gradually shifted, particularly in East and West Africa, following the introduction of DENV-3 and DENV-1. These serotypes were associated with major outbreaks, including DENV-3 outbreaks in Senegal in 2009 and Kenya and Tanzania in 2017, as well as DENV-1 outbreaks in Tanzania in 2019. These consecutive outbreaks, especially in East Africa, were caused by independent introductions of DENV-1 and DENV-3 from upper Southeast Asia for DENV-1 and Western Asia for DENV-3 [20,21].

The change of serotype has not been equal in all areas with the DENV. In certain countries, serotype shifts have been fairly gradual and predictable, whereas in others they have been much more sudden and unpredictable, and these changes have often been accompanied by shifts in the epidemiological situation. To illustrate, in Latin America, while DENV-2 was dominant for several decades, it was slowly replaced by DENV-3 in the 2000s, leading to a major epidemic in the Caribbean and Central America [22]. This was accompanied by a significant increase in hospital admissions and deaths due to severe dengue. The early 2000s reintroduction of DENV-4 into the Americas, after a long absence, led to widespread outbreaks in countries such as Cuba and Puerto Rico, where populations had little or no immunity to this serotype [23]. The reappearance of DENV-4 in these areas also emphasized the risks of serotype changes in naive populations, as well as the need to vigorously monitor and intervene to prevent outbreaks of serotype changes. A compilation of a few well-documented changes in DENV serotypes is summarized in Table 1. Overall, serotype switching appears to be a dynamic but heterogeneous process across dengue-endemic regions. While replacement events frequently involve DENV-1 and DENV-2, recent shifts toward DENV-3 and DENV-4 have also been documented in several countries. Nevertheless, in hyperendemic settings, multiple serotypes can co-circulate for prolonged periods before a dominant serotype emerges. These observations reflect a period of epidemiological equilibrium, during which competing viral populations coexist without a sufficient selective advantage for displacement until ecological or immunological conditions favor a particular serotype.

It is also interesting to note that although dengue serotype shift has frequently coincided with major dengue epidemics, available evidence showed that serotype switching is not always a prerequisite for outbreak occurrence. Recurrent outbreaks have also been documented during periods of stable serotype dominance. For instance, a stable DENV-1 dominance during the 2013–2015 outbreak period in Singapore was shown to coincide with climate factors [24].

**Table 1:** Summary of Major DENV Serotype Shifts Across Regions.

Region/Country	Year(s) of Shift	Serotype Replaced → Dominant	Outbreak Magnitude/Severity	Key Observations	Reference
Central India	2019–2023	DENV-1 → DENV-2	Outbreaks with moderate severity	Novel genotype emergence; displacement of prior serotype	[13]
Singapore	2007	DENV-1 → DENV-2	Large outbreak; increased hospitalizations	Clade replacement within DENV-2 coincided with the outbreak	[15]
Singapore	2013	DENV-2 → DENV-1	Major outbreak	DENV-1 rapidly displaced DENV-2 with the emergence of a new lineage of genotype III	[16]
Bangladesh	2019	DENV-1/DENV-2 → DENV-3	The largest recorded outbreak at that time	DENV-3 continued to dominate subsequent surges	[17]
Lao PDR	2016–2018	DENV-1 → DENV-4	Countrywide upsurge	Follows two prior large epidemics	[18]
Thailand	2004–2008	DENV-1 → DENV-4 → DENV-1	Significant outbreaks	Rapid displacement highlights dynamic serotype turnover	[19]
Tanzania, Africa	2017–2019	DENV-3 →DENV-1	One smaller outbreak and one major epidemic	DENV-1 was likely imported into the country, where the lack of prior circulation resulted in widespread transmission and explosive outbreaks among a susceptible population	[20]
Latin America	2000s	DENV-2 → DENV-3	Major outbreaks, increased hospitalizations & deaths	Gradual replacement over several years	[22]
Americas (Suriname, Mexico, Puerto Rico, and El Salvador)	1980’s	Absent → DENV-4	Widespread outbreaks	Populations largely naive; serotype introduced after prolonged absence	[23]
West Bengal, India	2020–2021	DENV-2 → DENV-3	Increased mortality and morbidity due to an increase in severe dengue cases	Re-emergence of DENV-3 associated with severity	[25]
Sri Lanka	2009	DENV-2/DENV-3 → DENV-1	Spike in hospitalizations/fatalities	Replacement associated with higher DHF/DSS	[26]
São Paulo, Brazil	2024	DENV-2 → DENV-1	Epidemic captured during vaccine evaluation	Demonstrates serotype-specific vaccine performance influence	[27]
Mexico	2022–2024	DENV-2 → DENV-3	Moderate outbreaks	Shift coincided with environmental changes influencing vector dynamics	[28]

## 5. Drivers of DENV Serotype Shift

The phenomenon of serotype shifts in dengue transmission is complex and depends on a variety of biological, environmental, and sociological factors. The combination of these factors shapes the dynamics of serotype circulation and may either accelerate or slow the process of serotype shift. Building on this, knowledge of the causes of serotype shift is important for alerting to outbreaks, implementing better surveillance, and establishing effective control interventions.

### 5.1. Host Immunity Dynamics

An immunological environment of the population is a key predictor of the dynamics of DENV serotypes. The coverage of immunity to DENV in the population is not always evenly distributed. Geographically driven by exposure history, age structure, human mobility and timing of outbreaks, heterogeneity in exposure history generates a patchwork of localized immune landscapes. In certain communities, high herd immunity to specific dengue serotypes suppresses their transmission, whereas neighboring communities with lower population immunity remain susceptible, allowing those serotypes to continue circulating. Notably, this patchwork shapes the overall level of herd immunity in the population, which builds over time as serotypes are distributed. This spatially heterogeneous immunity, coupled with the dynamics of vectors and movement of populations, has been demonstrated to cause gradual changes in serotype dominance at regional and national levels [29].

A major study revealed that DENV serotype shifts are heavily dependent on the accumulation of population-level immunity against the circulating serotype [30]. The higher the immunity, the lower the effective reproduction number (R) of that serotype, reducing its potential to transmit infection and creating ecological space for the emergence and spread of a different serotype. These changes are not just viral oscillations. They are a complex interplay among host immunity, viral transmission dynamics, and demographic factors (birth rates and population density) [31].

**Figure 2:**
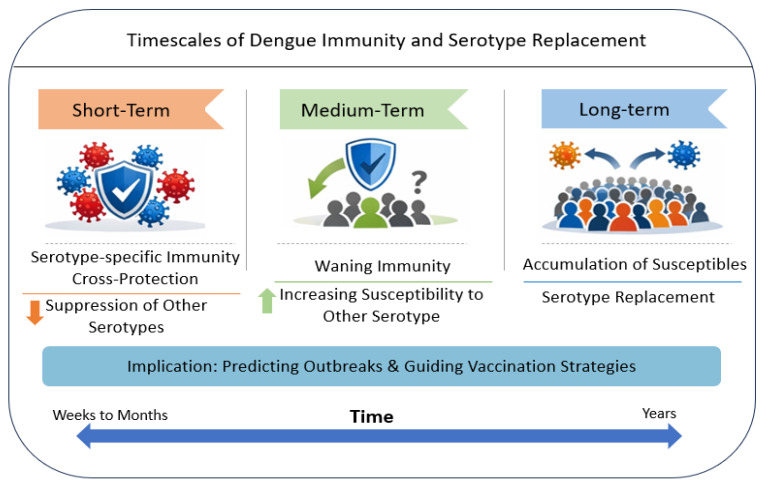
Timescales of Dengue Immunity and Serotype Shift.

Figure 2 displays the timescales of dengue immunity and how these immunity timescales influence the dynamics of serotypes and the vaccination strategies after infection with a specific serotype. A strong yet short-term immunity most efficiently develops to reinfect with the same serotype and provide temporary cross-protection against other serotypes. In fact, early experimental studies involving human volunteers indicated the principle of homologous immunity, which has been known for more than 70 years [32]. This is a period of temporary cross-immunity that inhibits the spread of other serotypes in the population. Immunity decreases gradually over the medium term, making an individual susceptible to other serotypes. At this stage, people who were once immune to the infection are exposed, allowing the spread of heterologous serotypes. In the long term, the presence of susceptible individuals throughout the population preconditions a serotype shift, with previously suppressed serotypes emerging and becoming dominant, thereby altering the balance of power in the competitive environment among circulating viruses [33]. A cohort study in Bangkok estimates that waning of cross-protection occurs within one to three years [2]. A mathematical modelling study also supported the transient nature of heterotypic immunity, estimating that complete cross-protection may persist for only one to two weeks [34]. Although the estimated duration varies between studies, both findings reinforce that heterotypic immunity is temporary rather than lifelong.

It is imperative to appreciate the dynamics of time to plan for the health of the community. Short-term cross-protection effects affect the immediate outbreak potential. Meanwhile, medium-term waning immunity helps explain when and in which order sequential serotype epidemics occur. Moreover, long-run susceptibility trends provide important guidance on vaccination strategies, including who should be targeted and when to initiate immunization campaigns.

In a different context, secondary infections with an alternative serotype have been demonstrated to be a risk factor for severe dengue, especially through antibody-dependent enhancement (ADE), which magnifies the effects of a dengue serotype shift [35,36]. In ADE, non-neutralizing or sub-neutralizing antibodies generated by the primary infection bind to the heterologous virus without inactivating it. These complexes promote viral replication and dysregulated immune responses, which put individuals at risk of unstable clinical responses. In addition to its clinical significance, ADE might also affect the dynamics of serotype at a population level. Secondary infections may result in increased viremia by greater viral replication in the body and increasing the rate of infection in mosquitoes and their subsequent transmission [37,38]. This mechanism may be a form of selective advantage in populations with high heterotypic immunity levels. As a result, it can enhance the growth and ultimate dominance of serotypes that can take advantage of ADE-mediated immunity.

Together, the combination of cumulative homologous herd immunity, short duration of cross-protective immunity, and the enhancement of heterologous infections by ADE contributes to the formation of DENV serotype competition. These interlinked immunological events provide a consistent model for explaining how a particular serotype can gradually decrease and be replaced by another, driving the circular nature of serotype predominance observed in an endemic situation.

### 5.2. Vector Ecology and Mosquito Population Dynamics

The distribution and abundance of mosquito vectors, predominantly *Aedes aegypti* and *Aedes albopictus*, are critical factors contributing to DENV serotype shifts [39]. Changes in the transmission efficiency of various serotypes can occur as a result of changes in the dynamics of the population of vectors, caused by variations in the environment, season or control actions. The differences in the competence and preference of vectors can influence the preeminence of the serotypes that circulate in regions with multiple serotypes. Indicatively, an Australian study revealed that both *Ae. aegypti* and *Ae. albopictus* had a significant variation in the rates of infection among different serotypes, with higher susceptibility to DENV-1 and DENV-2 than to other serotypes [40].

Geographic variation also affects the susceptibility and the potential for mosquito transmission. Traditional experimental studies indicated that mosquito population density varies across regions in terms of acquiring and transmitting different strains of DENV [41]. Subsequent research emphasized effects at the genotype level, namely the Southeast Asia genotype (which is now called Asian/American) of DENV-2 that was observed to spread more effectively in mosquitoes and had also a shorter extrinsic incubation period (EIP) as compared to the American genotype, amplifying its epidemic potential [42]. Likewise, a study has suggested that competition between DENV serotypes in the mosquito midgut during co-infection can significantly alter the potential to transmit, in this case, favoring the ability of DENV-1 to be competitively displaced by DENV-4 [43].

The capacity of a mosquito to acquire, replicate, and transmit a virus is known as vector competence, which varies by serotype and mosquito genotype [44]. Such variability can be one explanation for the discrepancies between human and mosquito infection patterns. As an example, even though DENV-4 dominated human infections during the study period in Brazil, DENV-1 and DENV-3 were more commonly detected in mosquitoes, indicating silent maintenance of some serotypes during epidemics. This also highlights that the dynamics of transmission are influenced by the rate of infection by vectors, the host immunity, and the fitness of the virus [45].

Serotype dynamics could also be molded by co-infection and competition among mosquitoes. Simultaneously exposing *Ae. aegypti* to multiple DENV serotypes has resulted in competitive interference, with some serotypes being preferentially transmitted depending on the genotype of the vectors and the fitness of the viruses [46]. Correspondingly, these kinds of interaction can be used to explain the negligent persistence of minority serotypes in the populations of vectors before they become dominant during the next epidemic.

There is also the contribution of behavioral ecology towards the serotype dynamics that are driven by vectors. Generally, *Ae. aegypti* is the more efficient DENV transmission vector since it is strongly linked to human habitats. Conversely, *Ae. albopictus* is capable of maintaining the transmission of DENV in peri-urban and rural regions, which expands the ecological niche of the virus [47]. Variations in resting, feeding, and oviposition behavior have an impact on the population density and contact with humans and hence on serotype-specific transmission. An example is that the endophilic behavior of *Ae. aegypti* is concentrated in urban areas, which may favor serotypes circulating in human populations and possibly better adapted to serotype requirements in the host.

In line with this, interventions that change the population of mosquitoes, such as spraying with insecticides, manipulating the environment, or releasing genetically modified mosquitoes, can indirectly affect serotype dynamics. These strategies can alter the relative transmission success of alternative DENV serotypes and could affect outbreak patterns. Indicatively, Wolbachia-infected *Ae. aegypti* are less competent to DENV-1 and DENV-2, which may selectively change the serotype distribution in areas where releases are performed [48].

### 5.3. Viral Evolution and Genetic Diversity

Most ribonucleic acid (RNA) viruses, including DENVs, have high mutation rates since the RNA polymerase of the virus lacks proofreading activity. This constant production of mutations generates significant genetic diversity within and among serotypes, which is the raw material of evolutionary change and contributor to serotype shift [49]. Among serotypes, distinct genotypes may evolve, and sometimes with different transmission efficiency, virulence or epidemiological fitness. This allows one lineage to outcompete another in case of a change in ecological or immunological conditions.

The most pronounced aspect of DENV evolution is the purifying selection, which is predominant throughout the viral genome. This is a process of evolution whereby unhealthy mutations will be eliminated in the population. Moreover, the frequency of synonymous mutations is much higher than that of nonsynonymous mutations. This indicates strong constraints on the amino acid substitutions tolerated to preserve functional proteins during alternating replication in mosquitoes and humans. Comparative studies of DENV-2 have presented lineage-specific selection pressures, especially in the E gene, demonstrating the heterogeneous nature of viral adaptation across geographies and time [50].

Adaptive evolution takes place in major antigenic areas, again targeting the E protein, with mutations having the potential to diminish recognition through neutralization of antibodies. These immune-escape mutations are a selective advantage in those populations with already existing immunity, allowing other serotypes that have previously been only minor serotypes to emerge. At the same time, amino acid changes in DENV-2 E proteins have been reported in studies in Vietnam, coinciding with the increase of these serotypes during epidemic cycles, with a direct correlation between immune-mediated evolution and serotype shifts [51].

Stochastic processes also influence viral evolution and serotype shifts. Intrinsic fitness is irrelevant since random events can have an effect on the persistence or dominance of the viral lineages obtained. For instance, genetic drift may lead to changes in the allele frequencies of small viral populations at early outbreak seeding or seasonal population decreases. Population bottlenecks in mosquitoes, in which only a subset of ingested viral particles become infectious and are transmitted to the salivary glands, can dramatically alter the number of viruses transmitted [41]. The introduction of a new serotype through travel or migration can result in founder effects, even in human populations that may not be more fit than the local strains. Additionally, temporal windows of increased susceptibility to chance spread of certain variants are caused by seasonal changes in the populations of vectors and hosts [52]. These random forces work together with selection. That is, a profitable mutation might not be able to establish itself, whereas a neutral or even less fit variant may gain prevalence.

The complex interaction of mechanisms creates serotype shift through a combination of genetic diversity, purifying and adaptive selection, immune-driven evolution, and stochastic processes in the context of changing host immunity. The high mutation rate results in viral variants, and the maintenance of functional stability through purifying selection, along with adaptive mutations, especially those necessary to escape the immune system, will enable one serotype to outcompete the others. The variants that survive are determined by stochastic events, and transient opportunities to grow arise due to changes in host immunity. This suggests that viral evolution, driven by deterministic and probabilistic forces, is necessary to explain the cyclical patterns of serotype predominance observed in endemic regions.

### 5.4. Climate Change and Environmental Factors

The environmental conditions are also critical in influencing the ecology and competence of mosquito vectors to shape DENV serotype transmission. The *Ae. aegypti* and *Ae. albopictus* are very sensitive to temperature, rainfall, humidity, and wind, which, in combination, determine their development, survival, and ability to transmit [53,54,55]. Higher temperatures promote the development of mosquitoes and enhance survival, with optimum conditions observed in the range of 25–30 °C, thereby increasing transmission efficiency [56,57,58]. On a similar note, rainfall and humidity have a modulating effect on the abundance of vectors. While heavy rainfall and long wet seasons provide breeding habitat for larvae, excessive rainfall can temporarily reduce populations, though it also encourages the development of their breeding habitats. Following this, high humidity increases mosquito longevity by alleviating desiccation stress and maintaining transmission persistence [59,60]. All these factors affect the coexistence of multiple serotypes, intensifying competition and increasing the likelihood of a serotype shift.

In both endemic and previously unaffected areas, climate change, along with land-use change and urbanization, is expanding the geographic and temporal distribution of Aedes mosquitoes, potentially increasing the frequency and duration of dengue transmission [61,62,63,64,65]. These are further enhanced by urban heat islands and poor water management systems, which create a favorable microenvironment that supports the proliferation of vectors.

Although environmental and climatic factors are evident in only moderating the transmission dynamics, their direct role in determining serotype shifts remains more poorly understood. When the environmental conditions are conducive to transmission, herd immunity can be overcome by high vector efficiency [66]. Climate variability, such as the El Niño Southern Oscillation, affects dengue outbreaks by altering the abundance of vectors and reducing the EIP, which may contribute to changes in serotype prevalence [67,68].

The recent outbreaks, including those in Latin America and Mexico, illustrate that period of enhanced transmission, driven by environmental variability, can coincide with shifts in the circulation of serotypes [69]. Still, these associations should be taken with a grain of salt, as they do not prove a direct causal relationship. In general, climate and environmental change can be viewed as modulators of dengue transmission, which may indirectly shape serotype dynamics rather than as direct drivers of serotype shifts.

### 5.5. Population Dynamics and Socioeconomic Factors

The transmission of dengue is closely related to some fundamental demographic processes that continually redefine the list of people who are vulnerable in a given community. The constant influx of new, immunologically naive individuals is factor that allows dengue to persist and cause cyclic epidemics over several years. Meanwhile, immunity is disproportionately developed in certain age groups. Seroprevalence studies have all indicated that the levels of dengue exposure by age increase. The immunity is unevenly distributed across the age groups, with the seroprevalence starting to increase at 40.0–70.0% in children below the age of 10 years [70]. It also reaches about 80.0–90.0% in adults in highly endemic settings, indicating repeated lifetime exposure to various serotypes [71]. Therefore, children and younger populations carry a larger share of infections during outbreaks, as susceptible individuals are concentrated in these age groups.

These dynamics are enhanced by urban growth. Overcrowding, poor housing, and water storage practices create ideal breeding grounds for mosquitoes, and even small changes in the environment or immunity can trigger sudden shifts in circulating serotypes, leading to larger and faster outbreaks [72,73]. The socioeconomic differences further compound the situation; poorer communities tend to have more contact with mosquitoes [74]. This results in more frequent infections and a faster buildup of immunity against locally circulating serotypes. Economic differences also impact the time and identification of serotype shifts. Regions with poor surveillance or limited health care access might underreport cases, resulting in a lag in the acceptance of new serotypes. This has the potential to delay a public health response, providing a novel serotype with a competitive edge in achieving circulation.

Human movement is like a thread that connects various populations. The virus can also be transported by short trips, commuting, and seasonal migration to new areas where immunity is weak, occasionally allowing a new serotype to establish itself and spread rapidly [75]. Migration and population mixing on a long-term basis also reshape the immunity landscape, making pockets of susceptibility that can sustain dengue virus transmission over time. Nevertheless, movement alone seldom causes serotype shifts, as it interacts with mosquito abundance, weakening immunity and viral characteristics that allow some lineages an advantage [76].

The ability to concurrently comprehend the interplay of population growth, immunity, urbanization, mobility, and health system capacity can keep the public health authorities one step ahead. By anticipating where and when outbreaks are likely, prioritizing surveillance and lab resources, and focusing vector control on the most vulnerable communities, the authorities can ease the burden of dengue and better protect the most vulnerable population.

## 6. Impact of Dengue Serotype Shift

### 6.1. Impact on Disease Severity

Epidemiological data indicate that shifts in dengue serotype are closely linked to increased disease severity. When a new serotype replaces a previously dominant one, populations with preexisting immunity to other serotypes are particularly susceptible, often leading to an increase in the prevalence of severe manifestations of dengue, such as hemorrhage, shock and multiorgan failure.

The relationship is depicted by the historical outbreaks. In Bangladesh, the re-emergence of DENV-3 after some years of DENV-2 domination coincided with a significant increase in severe cases over the following years [77]. On the same note, in 2009, in Sri Lanka, when DENV-1 was replaced by DENV-2, the hospitalization and death rates shot up dramatically [26].

Prospective cohort studies support the change of serotypes with severity through the interaction of circulating serotypes and host immune history. Secondary infections with recently spread DENV-2 and DENV-4 strains were related to increased rates of severe disease compared to infections by previously dominant serotypes [78,79]. In comparison, DENV-1 and DENV-4 were analyzed in periods of serotype shift in Brazil, and distinct patterns of warning signs and laboratory abnormalities were observed with replacement events potentially changing the clinical profile of dengue in populations [80].

These results demonstrate that serotype changes should not be considered a simple virological phenomenon but rather a primary driver of epidemiological and clinical processes. Additionally, the emergence of a new dominant serotype can increase disease severity, especially in populations with partial resistance to prior exposure to other serotypes.

### 6.2. Impact on Outbreak Preparedness and Response

Serotype shifts may seriously affect preparedness and response measures for dengue epidemics. Epidemiological studies also indicate that serotype turnover can immediately result in explosive epidemics, increase the burden on healthcare and change the nature of epidemics. The increase in hospitalizations, disease severity, and changes in the clinical profile of affected groups are often associated with shifts in circulating serotypes [79]. Collectively, these results support the argument that serotype shift is a factor in the scale and clinical complications of dengue outbreaks, influencing the timing, magnitude, and health impact of dengue epidemics on populations.

Therefore, the response plans to outbreaks prepared by public health authorities should be flexible and adaptable, and take into account the potential for serotype shifts. This involves having adequate hospital capacity, training healthcare workers to identify severe dengue cases, and having rapid response mechanisms in place, such as targeted vaccination or vector control campaigns.

### 6.3. Impact on Vaccines and Sylvatic Risk

Vaccination against dengue poses special challenges due to the intricate interplay between the epidemiology and the biology of the virus. Dengue immunity is mostly serotype-specific; that is, immunity to one serotype does not necessarily extend to another. Partial immunity to a different serotype may, in some instances, increase the risk of ADE, especially when the seronegative state occurs during vaccination.

This ever-changing trend of circulating serotypes also makes it difficult to predict vaccine performance. Effective vaccines must give balanced protection against all four serotypes as cross-protection is incomplete. Furthermore, viral evolution and divergent lineage emergence might be factors that affect vaccine effectiveness, as different viruses may respond differently to different vaccines. An additional challenge may be contributed by sylvatic DENVs spillover in human. These viruses are genetically different. The existing vaccines might not provide complete protection, as evidence suggests that cross-neutralization is limited [81,82], an important gap in current vaccine testing.

Despite these additional factors, we still lack a comprehensive understanding of the effectiveness of vaccines against each serotype. Follow-up in clinical trials and real-world studies is often limited in duration, and in some cases, only a few cases of some serotypes are available. This complicates the ability to draw solid conclusions about vaccine interactions with various serotypes.

Nonetheless, there is good news amid these challenges. More recent dengue vaccines, such as TAK-003, have demonstrated good safety profiles and can protect against hospitalization across different dengue-prone areas. The findings of the Phase 3 trials are supported by real-world evidence of the 2024 outbreak in Brazil, although protection by serotype was varied. Indicatively, in a period of DENV-1 preeminence, protection against DENV-1 (around 74%) was lower than that of DENV-2 (around 98%) despite the overall effectiveness of the vaccine in symptomatic dengue prevention being approximately 80% [27]. This demonstrates that alterations in circulating serotypes can affect vaccine performance and that continued monitoring is important.

There is also a need to plan the implementation of dengue vaccination programs. It is important to match the right vaccine with the right population, follow up on those who have already received exposure, and administer the vaccine at the time of high risk. Building on this, post-licensure surveillance is necessary to identify that vaccines are functioning as intended, identify any safety issues, and enable strategies to be modified, where necessary. A combination of vaccination and data on seroprevalence, along with targeting a specific age group, can enhance both safety and efficacy. Flexibility in responding to real-time information on outbreaks and the serotypes currently active in the area is of great importance in regions where dengue is widespread.

This emphasizes the necessity of integrating clinical, virological, and genomic surveillance to ensure that both urban and sylvatic dengue cycles are monitored. There should be adaptive strategies of vaccination, considering the local patterns of serotype, population immunity, and new information about the genetics of viruses. The fact that there are chances of sylvatic spillover events, and in particular in areas where there are environmental changes, reminds us that the effectiveness of vaccines transcends beyond the four classical dengue serotypes. In brief, dengue vaccines will be an important tool in controlling the disease, although their long-term effects will depend on further evaluation and a better understanding of the impact of viral diversity on immunity.

## 7. Knowledge Gaps and Future Direction

Although knowledge of the dynamics of dengue serotypes has improved, significant gaps remain that can only be addressed by further research. The exact mechanisms driving serotype shifts, particularly during unprecedented outbreaks, are not fully understood, including how viral, immunological, environmental, and vector factors interact to favor one serotype over another. Viral mutations that influence immune evasion and transmission, as well as the role of vector and environmental dynamics in facilitating serotype shift, require further investigation. In the short term (1–3 years), priority should be given to integrated serotype and genotype-based surveillance and enhanced post-vaccination monitoring, capturing serotype- and genotype-specific vaccine effectiveness and adverse events to inform timely interventions. Furthermore, surveillance and early warning systems need refinement, including integration of genomic data and neighborhood-level monitoring to improve artificial intelligence (AI)-driven outbreak forecasting. At the same time, rapid diagnostics for serotype identification, especially broad-coverage assays, remain essential. Over the medium term (3–5 years), research should focus on mapping the immune landscape to understand population-level immunity and cross-protection. During this era, vaccine development faces further challenges since currently used vaccines must be effective in broad protection against all four serotypes and emerging strains, with consideration of already existing immunity due to previous natural infections. The optimization of formulations, booster schedules, and alternative or combination vaccine platforms is crucial in areas with a high rate of serotype changes. Long-term (>5 years), real-time predictive modeling that incorporates climate, genomics, vector ecology, and immunology can enhance outbreak prediction and alert, with genomic surveillance and open-data sharing able to track virus evolution, emergent genotypes, and the effect of control measures on serotype dynamics.

## 8. Conclusions

The drivers of serotype shift are multifactorial and interconnected, involving complex interactions. Although the five mechanisms mentioned are biologically plausible, the relative contributions of each driver are not well known and may differ across contexts. The reviewed studies provide strong empirical evidence supporting context-dependent immunogenic serotype shifts, whereas the contributions of viral evolution and vector ecology appear to vary more substantially between settings. At the same time, observed correlations between the climate changes and human factors with serotype shifts in several cases might reflect an indirect effect rather than a direct cause-and-effect relationship. These doubts underscore the need to exercise caution in interpretation and to consider integrating various lines of evidence. Overall, by understanding the primary drivers of serotype shifts, public health authorities can implement more effective surveillance, prevention, and response strategies, ultimately reducing the public health burden of dengue.

## Data Availability

Data sharing does not apply to this article, as no new data were created or analyzed.

## Supplementary Materials

The following supporting information can be downloaded at: https://www.mdpi.com/article/10.3390/v18060683/s1, Figure S1: Phylogenetic tree of DENV-1 demonstrating the diversity of five genotypes (I–V); Figure S2: Phylogenetic tree of DENV-2 demonstrating the diversity of six genotypes (I–VI); Figure S3: Phylogenetic tree of DENV-3 demonstrating the diversity of four genotypes (I, II, III and V). Genotype IV (not shown) has largely been replaced or is no longer widely circulating; Figure S4: Phylogenetic tree of DENV-4 demonstrating the diversity of four genotypes (I–IV).
