# Supplementary material for: Determinants of Dengue Serotype Shifts: A Narrative Multifactorial Perspective
Source: Viruses. 2026 Jun 18;18(6):683. doi: 10.3390/v18060683 (PMC13308309; doi:10.3390/v18060683)
Supplement: Supplementary file 1 [file viruses-18-00683-s001.zip › viruses-4338266-supplementary.pdf]

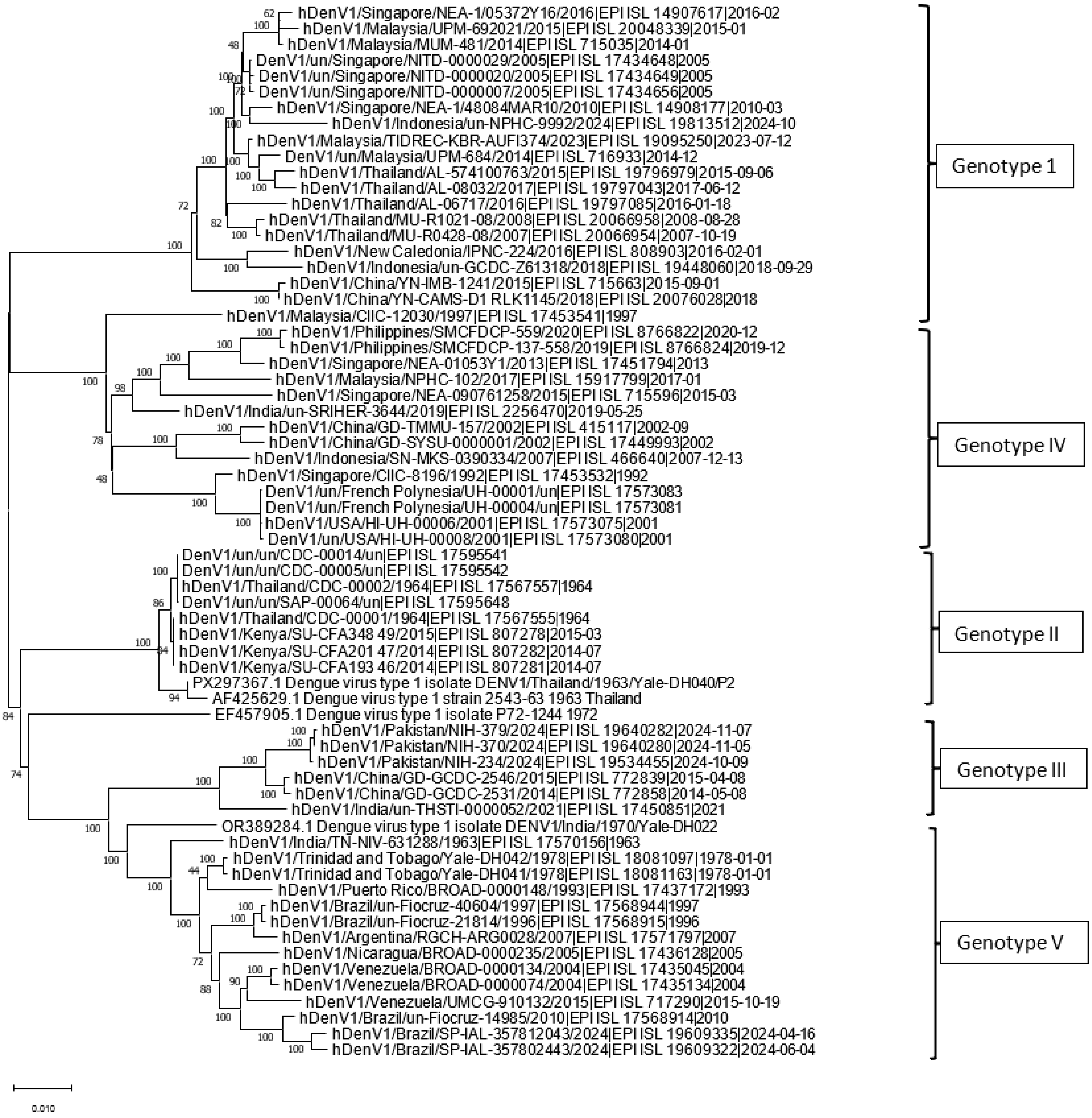


Figure S1: Phylogenetic tree of DENV-1 demonstrating the diversity of five genotypes (I-V)

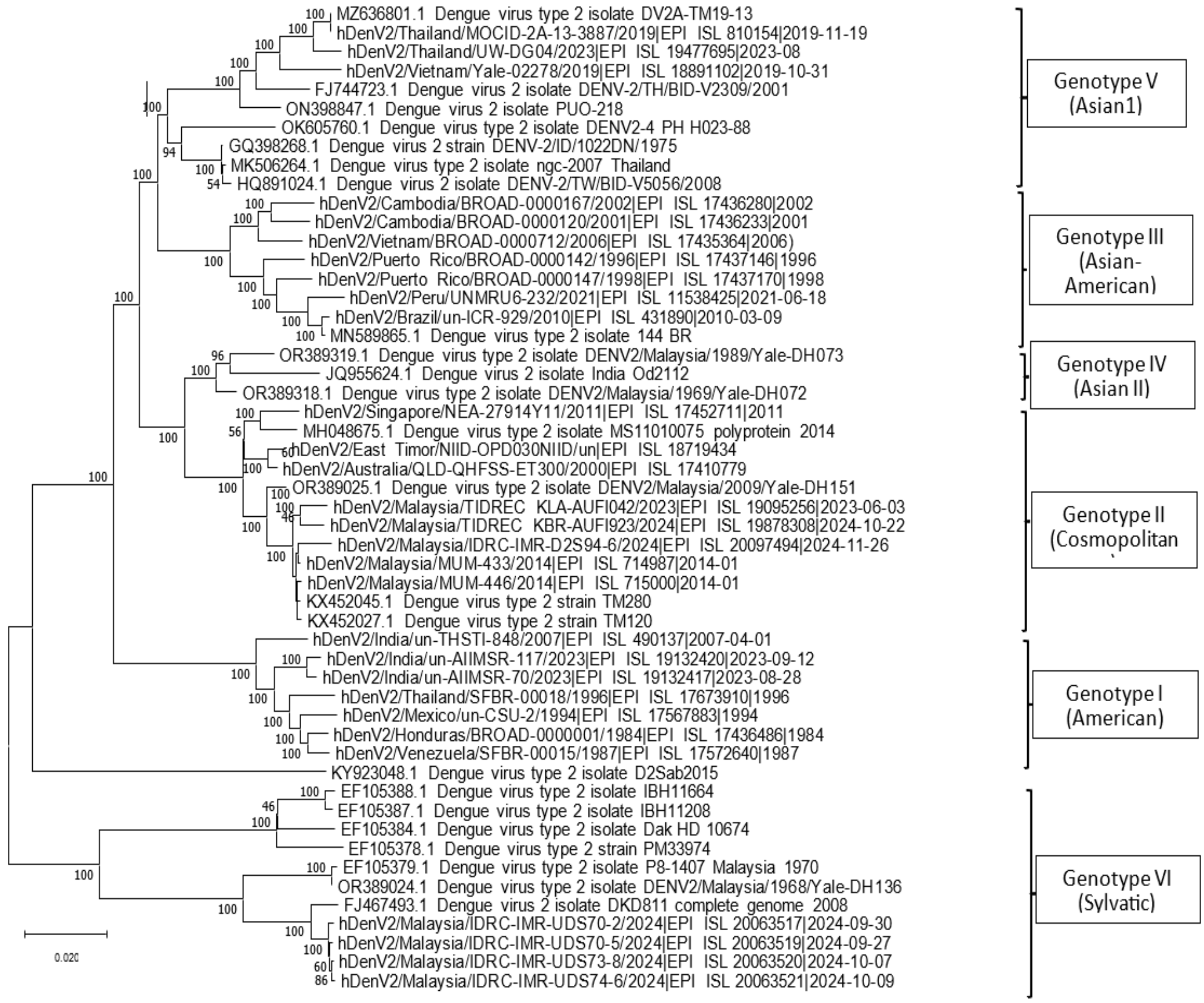


Figure S2: Phylogenetic tree of DENV-2 demonstrating the diversity of six genotypes (I-VI)

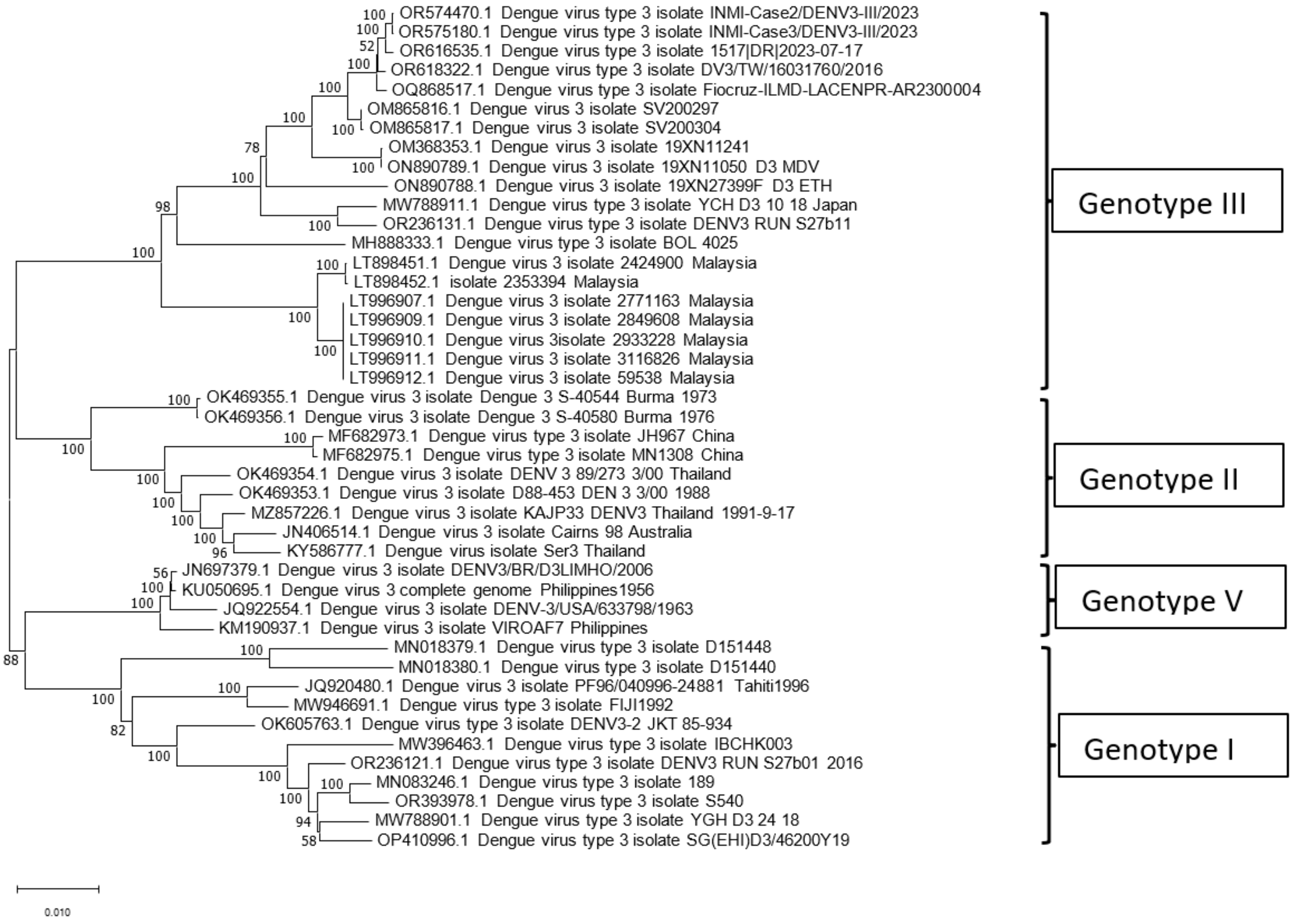


Figure S3: Phylogenetic tree of DENV-3 demonstrating the diversity of four genotypes (I, II, III and V). Genotype IV (not shown) has largely been replaced or is no longer widely circulating.

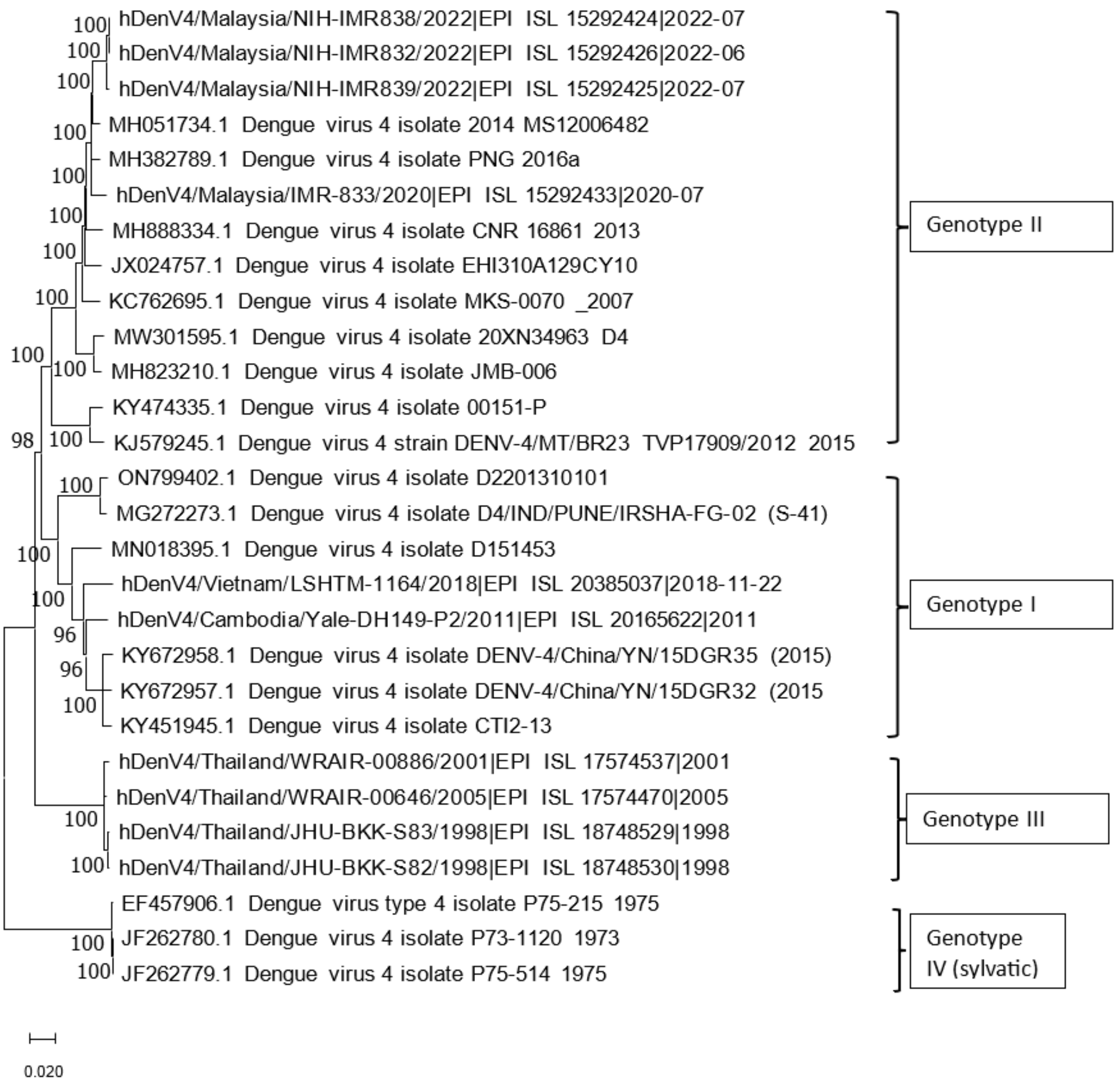


Figure S4: Phylogenetic tree of DENV-4 demonstrating the diversity of four genotypes (I-IV).
